# Supplementary material for: Psychological predictors of vaccination intentions among U.S. undergraduates and online panel workers during the 2020 COVID-19 pandemic
Source: PLoS One. 2021 Nov 30;16(11):e0260380. doi: 10.1371/journal.pone.0260380 (PMC8631617; doi:10.1371/journal.pone.0260380)
Supplement: S1 File — Study materials, supplementary measures and analyses. (DOCX) [file pone.0260380.s001.docx]

Online Supplemental Material for

**Psychological Predictors of Vaccination Intentions Among U.S. Undergraduates and**

**Online Panel Workers During the 2020 COVID-19 Pandemic**

ONLINE SUPPLEMENTAL MATERIAL FOR

PSYCHOLOGICAL PREDICTORS OF VACCINATION INTENTIONS AMONG U.S. UNDERGRADUATES AND ONLINE PANEL WORKERS DURING THE 2020 COVID-19 PANDEMIC

TABLE OF CONTENTS

[STUDY MATERIALS 3](#_Toc87195010)

[Main Measures 3](#_Toc87195011)

[Vaccination Intention: COVID-19 3](#_Toc87195012)

[Vaccination Intention: Dysomeria (Haase et al., 2019; Jolley & Douglas, 2014) 5](#_Toc87195013)

[5C Psychological Antecedents of Vaccination (Betsch et al., 2018) 6](#_Toc87195014)

[Vaccine Knowledge 7](#_Toc87195015)

[Past Vaccination Behavior (Flu Shot) 7](#_Toc87195016)

[Anti-Vaccine Conspiracy Beliefs (Jolley & Douglas, 2014) 7](#_Toc87195017)

[Perceived Vaccine Danger (Jolley & Douglas, 2014) 8](#_Toc87195018)

[Mistrust in Science/Scientists (Nadelson et al., 2014) 8](#_Toc87195019)

[Analytical Thinking (Norris & Epstein, 2011) 8](#_Toc87195020)

[Self-Other Overlap (Aron et al., 1992) 9](#_Toc87195021)

[Perceived Vulnerability to Disease (Duncan et al., 2009) 9](#_Toc87195022)

[Attention Check 10](#_Toc87195023)

[Demographics 10](#_Toc87195024)

[Supplementary Measures: Study 1 (Undergraduates) 11](#_Toc87195025)

[Open-Ended Questions 11](#_Toc87195026)

[Vaccinations Required & Recommended by University 12](#_Toc87195027)

[Empathic Concern (Davis, 1980) 13](#_Toc87195028)

[Delayed Gratification (Hoerger et al., 2011) 13](#_Toc87195029)

[Risk-Taking (Weber et al., 2002) 13](#_Toc87195030)

[Behavior Identification Form (Vallacher & Wegner, 1989) 14](#_Toc87195031)

[Supplementary Measures: Study 2 (Online Panel Workers) 15](#_Toc87195032)

[Open-Ended Questions 15](#_Toc87195033)

[COVID-19 Optimism Bias 15](#_Toc87195034)

[Reasons Influencing COVID-19 Vaccination 15](#_Toc87195035)

[Attitudes about Social Distancing 16](#_Toc87195036)

[Perceived Health/Economic Impact of COVID-19 16](#_Toc87195037)

[COVID-19 Media Consumption 17](#_Toc87195038)

[SUPPLEMENTARY RESULTS AND ANALYSES 18](#_Toc87195039)

[Study 1 Supplementary Analyses 18](#_Toc87195040)

[Attention Check 18](#_Toc87195041)

[Supplementary Variables Descriptive Statistics 18](#_Toc87195042)

[Main Variables Correlations 19](#_Toc87195043)

[Robustness Check for Time 20](#_Toc87195044)

[Study 2 Supplementary Analyses 20](#_Toc87195045)

[Attention Check 20](#_Toc87195046)

[Additional Demographics 21](#_Toc87195047)

[Main Variables Correlations 23](#_Toc87195048)

[Robustness Check with COVID meta-data 24](#_Toc87195049)

[Optimism Bias Regarding COVID-19 Infection and Symptoms 24](#_Toc87195050)

[COVID-19 Vaccination Intention (Continuous Measure) 25](#_Toc87195051)

[COVID-19 Vaccination Intention (Discrete Measure) 28](#_Toc87195052)

[Reasons Influencing COVID-19 Vaccination 29](#_Toc87195053)

[Social Distancing and Health/Economic Impact of COVID-19 29](#_Toc87195054)

[COVID-19 Media Consumption 30](#_Toc87195055)

[REFERENCES 31](#_Toc87195056)

# STUDY MATERIALS

Below, we report the main measures and supplementary measures we collected for both Study 1 (undergraduates) and Study 2 (online panel workers). Unless otherwise indicated, the main measures were identical across studies. The items within each scale were presented in randomized order.

For Study 1, the measures were presented in the following order: 5C, dysomeria vaccination intention, COVID-19 vaccination intention, attitudes toward vaccinations required/recommended by the university, vaccine knowledge, past vaccination behavior (flu shot), anti-vaccine conspiracy beliefs, perceived vaccine danger, analytical thinking, mistrust in science/scientists, empathic concern, self-other overlap, delaying gratification, perceived disease vulnerability, risk-taking, behavior identification form, and demographics questionnaire.

For Study 2, the measures were presented in the following order: COVID-19 vaccination intention, dysomeria vaccination intention, COVID-19 vaccine reasons, past vaccination behavior (flu shot), 5C, vaccine knowledge, COVID-19 media consumption, anti-vaccine conspiracy beliefs, perceived vaccine danger, analytical thinking, mistrust in science/scientists, self-other overlap, perceived disease vulnerability, COVID-19 optimism bias, attitudes about social distancing, perceived health/economic impact of COVID-19, and demographics questionnaire.

Upon completing the study, all participants in both studies were thoroughly debriefed (e.g., dysomeria being a fictitious disease).

## Main Measures

### Vaccination Intention: COVID-19

Study 1 (Undergraduates)

A recent outbreak of a respiratory illness caused by a novel coronavirus (COVID-19) is spreading rapidly. There have been hundreds of thousands of confirmed cases and thousands of deaths around the world. As of [Date _______], ________ confirmed cases in the United States have been reported, along with ________ deaths. The Centers for Disease Control and Prevention (CDC) confirmed on January 30, 2020 that the COVID-19 has spread between two people in Illinois, representing the first instance of person-to-person spread with this new virus in the United States.

Much is unknown about how COVID-19 spreads. Current knowledge is largely based on what is known about similar coronaviruses (e.g., SARS, MERS). Person-to-person spread is thought to occur mainly via respiratory droplets produced when an infected person coughs or sneezes, similar to how influenza and other respiratory pathogens spread. These droplets can land in the mouths or noses of people who are nearby or possibly be inhaled into the lungs. It is recommended to regularly wash one’s hands and disinfect hard surfaces as the virus could be transmitted through these routes by touching a surface or object that has the virus on it and then touching their own mouth, nose, or possibly their eyes. Symptoms can range from mild illness to severe illness and death and are accompanied by fever, cough, and shortness of breath.

*Source: Centers for Disease Control and Prevention*

There is no specific antiviral treatment for COVID-19, and there is currently no vaccine to protect against COVID-19. However, if such vaccine were made available to prevent infection, would you receive it? Please select only one of the following:

- I would not receive the vaccination even if it’s free ($0).
- I would receive the vaccination only if it’s free ($0).
- I would pay up to $10 to receive the vaccination.
- I would pay up to $25 to receive the vaccination.
- I would pay up to $50 to receive the vaccination.
- I would pay up to $100 to receive the vaccination.
- I would pay more than $100 to receive the vaccination.

Until March 15, 2020, the number of cases (19) and deaths (0) displayed to participants remained constant. Starting March 16, 2020, cases and deaths were updated every few days (Table S1).

Table S1. Number of U.S. COVID-19 cases and deaths displayed to Study 1 participants.

| **Date Displayed in Survey** | **COVID**  **U.S. Cases** | **COVID U.S. Deaths** | **Number of Respondents** | **Participation Dates** |
| --- | --- | --- | --- | --- |
| February 14, 2020 | 19 | 0 | 236 | 02/24/2020-03/15/2020 |
| March 16, 2020 | 3,487 | 68 | 11 | 03/16/2020-03/19/2020 |
| March 20, 2020 | 15,219 | 201 | 11 | 03/20/2020-03/23/2020 |
| March 23, 2020 | 33,404 | 400 | 2 | 03/24/2020 |
| March 24, 2020 | 44,183 | 544 | 3 | 03/24/2020-03/25/2020 |
| March 25, 2020 | 54,453 | 737 | 2 | 03/26/2020 |
| March 27, 2020 | 85,356 | 1,246 | 4 | 03/28/2020-03/29/2020 |
| March 30, 2020 | 140,904 | 2,405 | 5 | 03/30/2020-03/31/2020 |
| March 31, 2020 | 163,539 | 2,680 | 4 | 04/01/2020 |
| April 1, 2020 | 186,101 | 3,603 | 3 | 04/01/2020-04/02/2020 |
| April 2, 2020 | 213,144 | 4,513 | 1 | 04/02/2020 |
| April 4, 2020 | 239,279 | 5,443 | 1 | 04/05/2020 |
| April 6, 2020 | 330,891 | 8,910 | 1 | 04/07/2020 |
| April 7, 2020 | 374,329 | 12,064 | 2 | 04/07/2020-04/08/2020 |
| April 8, 2020 | 396,011 | 12,754 | 1 | 04/09/2020 |
| April 9, 2020 | 427,460 | 14,696 | 1 | 04/09/2020 |
| April 10, 2020 | 459,165 | 16,570 | 5 | 04/10/2020-04/14/2020 |
| April 14, 2020 | 579,005 | 22,252 | 4 | 04/15/2020 |
| April 15, 2020 | 605,390 | 24,582 | 4 | 04/16/2020 |
| April 16, 2020 | 632,548 | 31,071 | 1 | 04/17/2020 |
| April 17, 2020 | 661,712 | 33,049 | 6 | 04/17/2020-04/21/2020 |

Study 2 (Online Panel Workers)

A recent outbreak of a respiratory illness caused by a novel coronavirus (COVID-19) is spreading rapidly. The Centers for Disease Control and Prevention (CDC) confirmed on January 30, 2020 that COVID-19 has spread between two people in Illinois, representing the first instance of person-to-person spread with this new virus in the United States. On March 11, 2020, the World Health Organization (WHO) officially declared the outbreak a pandemic. There have been millions of confirmed cases and hundreds of thousands of deaths around the world. As of July 20, 2020: 3,698,161 confirmed cases in the United States have been reported, along with 139,659 deaths.

The virus that causes COVID-19 is thought to spread mainly from person to person through respiratory droplets produced when an infected person coughs, sneezes, or talks. These droplets can land in the mouths or noses of people who are nearby or possibly be inhaled into the lungs. Spread is more likely when people are in close contact with one another (within about 6 feet). Symptoms can range from mild illness to severe illness and death and are accompanied by fever, cough, and shortness of breath, among others. There is much more to learn about the transmissibility, severity, and other characteristics associated with COVID-19, and investigations are ongoing.

*Source: Centers for Disease Control and Prevention*

There is no specific antiviral treatment for COVID-19, and there is currently no vaccine to protect against COVID-19. However, if such vaccine were made available to prevent infection, would you receive it?

- I would not receive the vaccination even if it’s free ($0).
- I would receive the vaccination only if it’s free ($0); If I need to pay money, I would not receive the vaccination.
- I would pay money to receive the vaccination.

Please indicate the maximum amount of money that you would personally pay to receive the COVID-19 vaccination.

|  | **$0** | **$500** |
| --- | --- | --- |

| I would pay up to $___ to receive the  COVID-19 vaccination. | 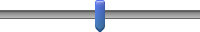 |
| --- | --- |

### Vaccination Intention: Dysomeria (Haase et al., 2019; Jolley & Douglas, 2014)

Please now imagine that you are the parent of an infant (Sophie, 8 months). Your doctor has provided you with the following information regarding the disease dysomeria and mentioned that there is a vaccination available.

Dysomeria: The DS-virus is a contagion spread by droplet infection. Early symptoms are fever and vomiting. Meningitis and impairment of motor and sensory functions are also common. In some cases, the DS-virus leads to permanent paralysis.

There is a vaccination against dysomeria. This vaccination effectively protects against infection and is highly recommended by the Centers for Disease Control and Prevention (CDC) for people of all ages. Adverse events such as fever, rash, restlessness and dizziness have been reported following 12% of all vaccinations (indicated by the darker rectangles in the graph below). In 88% of all cases, no side effects occurred (gray rectangles).


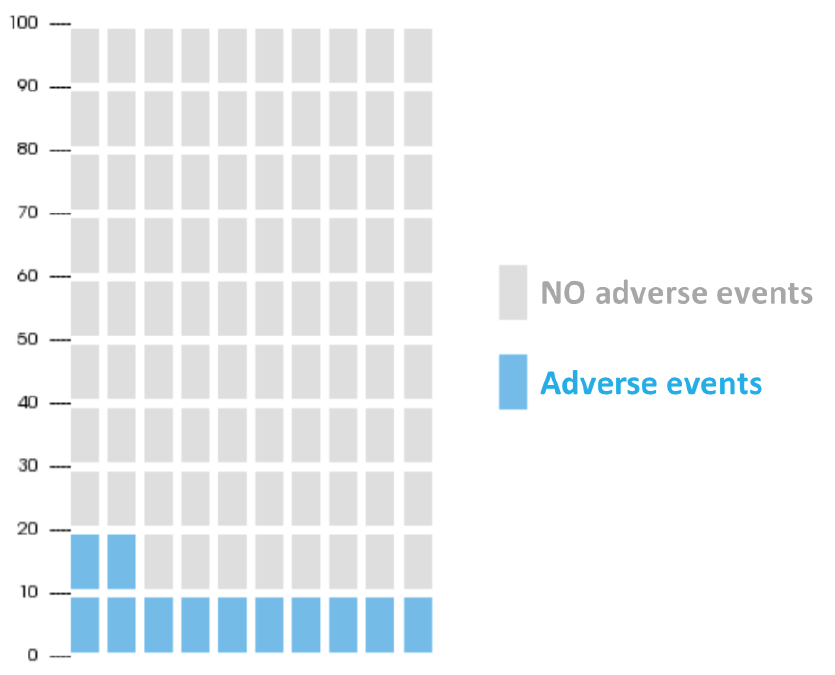


If you had the opportunity to vaccinate your child (Sophie, 8 months old) against dysomeria next week, what would you decide?

| 1=Definitely Not  Vaccinate | 2 | 3 | 4 | 5 | 6 | 7=Definitely Vaccinate |
| --- | --- | --- | --- | --- | --- | --- |

### 5C Psychological Antecedents of Vaccination (Betsch et al., 2018)

Please evaluate how much you disagree or agree with the following statements.

| Strongly Disagree | Moderately Disagree | Slightly Disagree | Neutral | Slightly Agree | Moderately Agree | Strongly Agree |
| --- | --- | --- | --- | --- | --- | --- |
| 1 | 2 | 3 | 4 | 5 | 6 | 7 |

Confidence

- I am completely confident that vaccines are safe.
- Vaccines are effective.
- Regarding vaccinations, I am confident that public authorities decide in the best interest of the community.

Complacency

- Vaccination is unnecessary because vaccine-preventable diseases are not common anymore.
- My immune system is so strong, it also protects me against diseases.
- Vaccine-preventable diseases are not so severe that I should get vaccinated.

Constraints

- Everyday stress prevents me from getting vaccinated.
- For me, it is inconvenient to receive vaccinations.
- Visiting the doctor’s makes me feel uncomfortable, this keeps me from getting vaccinated.

Calculation

- When I think about getting vaccinated, I weight benefits and risks to make the best decision possible.
- For each and every vaccination, I closely consider whether it is useful for me.
- It is important for me to fully understand the topic of vaccination before I get vaccinated.

Collective Responsibility

- When everyone is vaccinated, I don’t have to get vaccinated, too. (reversed)
- I get vaccinated because I can also protect people with a weaker immune system.^[[1]](#footnote-1)^
- Vaccination is a collective action to prevent the spread of diseases.

### Vaccine Knowledge

Compared to your peers, how much do you know about:

| 1=I am not at all knowledgeable | 2 | 3 | 4 | 5 | 6 | 7= I am extremely knowledgeable |
| --- | --- | --- | --- | --- | --- | --- |

- How vaccines work
- The benefits of vaccinations
- Real facts about vaccinations

### Past Vaccination Behavior (Flu Shot)

Study 1 (Undergraduates)

This past season, did you receive the flu shot? [Yes/No/Prefer not to answer]

Study 2 (Online Panel Workers)

This past flu season (October 2019−April 2020), did you receive the flu shot? [Yes/No]

### Anti-Vaccine Conspiracy Beliefs (Jolley & Douglas, 2014)

Please indicate your agreement using the following scale provided below.

| Strongly Disagree | Disagree | Somewhat Disagree | Neutral | Somewhat Agree | Agree | Strongly Agree |
| --- | --- | --- | --- | --- | --- | --- |
| 1 | 2 | 3 | 4 | 5 | 6 | 7 |

- Immunizations allow governments to track and control people.
- Vaccines are harmful, and this fact is covered up.
- Tiny devices are placed in vaccines to track people.
- Pharmaceutical companies, scientists and academics work together to cover up the dangers of vaccines.
- Vaccines are not tampered with. (reversed)
- The government is trying to cover up the link between vaccines and autism.
- Tiny devices are implanted in vaccines for the use in mind control experiments.

### Perceived Vaccine Danger (Jolley & Douglas, 2014)

Please indicate the degree to which you agree or disagree using the scale provided.

| Strongly Disagree | Disagree | Somewhat Disagree | Neutral | Somewhat Agree | Agree | Strongly Agree |
| --- | --- | --- | --- | --- | --- | --- |
| 1 | 2 | 3 | 4 | 5 | 6 | 7 |

- I feel uncertain about the potential side-effects of immunizations.
- I feel uncertain about the safety of immunizations.
- A large number of early vaccinations expose an infant’s immune system to avoidable risks.
- Multiple vaccines overwhelm the infant’s immune system.
- The side-effects of vaccinations are unforeseeable.
- Vaccines lead to allergies.
- I feel uncertain about the motives of those involved in immunizations (governments, pharmaceutical companies, etc.)
- Vaccinations cause the illness they are intended to protect against.

### Mistrust in Science/Scientists (Nadelson et al., 2014)

Please indicate your agreement on the five-point scale provided below.

| Extremely Unlikely | Unlikely | Neutral | Likely | Extremely Likely |
| --- | --- | --- | --- | --- |
| 1 | 2 | 3 | 4 | 5 |

- Scientists ignore evidence that contradicts their work.
- Scientific theories are weak explanations.
- We cannot trust scientists because they are biased in their perspectives.
- We cannot trust scientists to consider ideas that contradict their own.
- We cannot trust science because it moves too slowly.

### Analytical Thinking (Norris & Epstein, 2011)

| Completely False | Somewhat False | Neither True nor False | Somewhat True | Completely True |
| --- | --- | --- | --- | --- |
| 1 | 2 | 3 | 4 | 5 |

- I enjoy problems that require hard thinking.
- I am not very good in solving problems that require careful logical analysis. (reversed)
- I enjoy intellectual challenges.
- I prefer complex to simple problems.
- I don't like to have to do a lot of thinking. (reversed)
- Reasoning things out carefully is not one of my strong points. (reversed)
- I am not a very analytical thinker. (reversed)
- I try to avoid situations that require thinking in depth about something. (reversed)
- I am much better at figuring things out logically than most people.
- I have a logical mind.

### Self-Other Overlap (Aron et al., 1992)

Study 1 (Undergraduates)

Please select the picture which best describes your relationship with your acquaintances.

Study 2 (Online Panel Workers)

Please select the picture which best describes your relationship with members in your community.


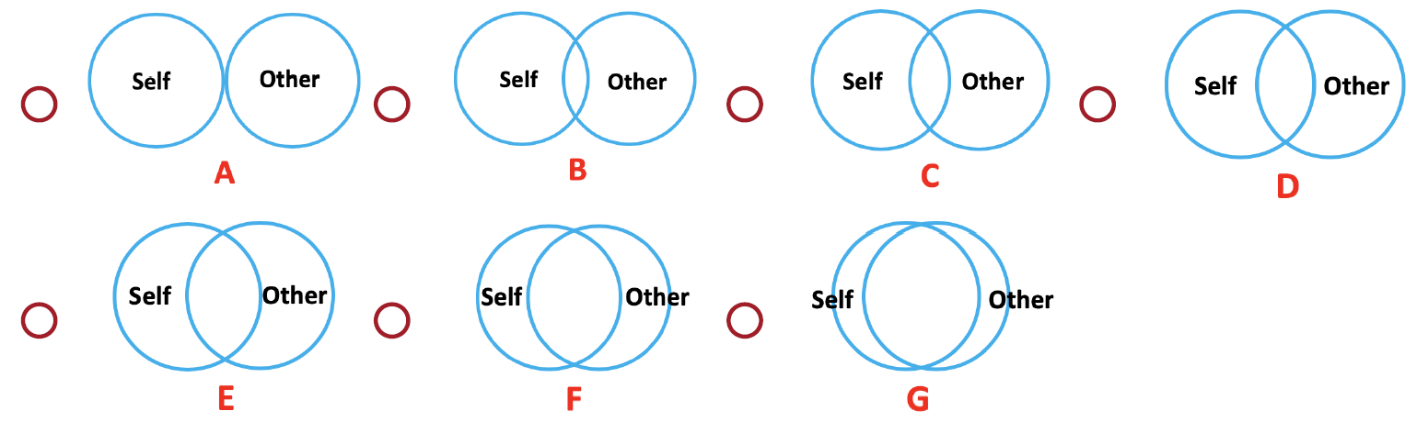


### Perceived Vulnerability to Disease (Duncan et al., 2009)

Rank the following phrases on a seven-point scale provided below.

| Strongly Disagree | Disagree | Somewhat Disagree | Neither Agree nor Disagree | Somewhat Agree | Agree | Strongly Agree |
| --- | --- | --- | --- | --- | --- | --- |
| 1 | 2 | 3 | 4 | 5 | 6 | 7 |

- In general, I am very susceptible to colds, flu and other infectious diseases.
- I am unlikely to catch a cold, flu, or other illness, even if it is 'going around.' (reversed)
- If an illness is 'going around,' I will get it.
- My immune system protects me from most illnesses that other people get. (reversed)
- I am more likely than the people around me to catch an infectious disease.
- My past experiences make me believe I am not likely to get sick even when my friends are sick. (reversed)
- I have a history of susceptibility to infectious disease.

### Attention Check

Study 1 and Study 2

For both Study 1 and Study 2, the following item was embedded in the perceived vaccine danger questionnaire:

- If you're reading this, please check "Strongly Agree."

Study 1 Only

For Study 1, the following item was asked prior to the demographics questionnaire:

- Thank you for your responses. You are almost finished with this study. Before answering a few questions about yourself, we have one last question. Do you think we should include your responses in our study? That is, did you take the study seriously and respond thoughtfully? Your credit assignment for this study does not depend on your response to this question. [Yes, I responded thoughtfully / No, I did not respond thoughtfully.]

Study 2 Only

For Study 2, the following item was embedded in the optimism bias measure:

| If you are reading this, please select 67 on  this sliding scale. | 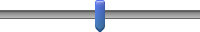 |
| --- | --- |

We decided to alter our attention check approach in Study 2 for three reasons. First, we noticed from our previous work that has used online panel workers that almost all participants claim that they responded thoughtfully even when we explicitly tell them that their payment will not be affected on the basis of their response. Thus, we decided to drop this particular question which did not seem to be providing much information about actual attentiveness. Second, for a question with two response options (“Yes, I responded thoughtfully” vs. “No, I did not respond thoughtfully”), the chance of answering “Yes” is 50% even if a participant were to select a random response. With a slider scale like above, there is only 1% chance that a non-attentive respondent will accidentally move the slider to the correct number. To ensure obtaining quality data, we decided to have a stricter attention check item in Study 2. Third, there were other measures (including the main COVID-19 vaccination intention item) that used a slider scale in Study 2, whereas all questions were in multiple-choice or Likert-type scale formats in Study 1. An ancillary reason for using the slider scale as an attention check was to confirm that participants were able to indicate their desired response using the slider format.

### Demographics

Study 1 and Study 2

What is your gender? [Male, Female, Prefer not to say, Other]

What is your age?

What is your ethnicity? [Asian/Asian American, Black/African American, Hispanic/Latino(a), Native American/Pacific Islander, White/European American, More than one, Prefer not to say, Other]

Study 2 Only

Have you previously been tested for COVID-19? [Yes, No, Prefer not to answer]

You indicated in the previous question that you were previously tested for COVID-19. If you feel comfortable with sharing the results, please indicate if you tested positive, negative, or prefer not to answer. [Positive Negative, Prefer not to answer]

Are you a parent? [No, Yes]

What state do you reside in?

Approximately, what is your household’s annual total income?

- $0−$25,000
- $25,001−$50,000
- $50,001−$75,000
- $75,001−$100,000
- $100,001−$125,000
- $125,001−$150,000
- $150,001−$175,000
- $175,001−$200,000
- More than $200,000

## Supplementary Measures: Study 1 (Undergraduates)

### Open-Ended Questions

Participants responded to the following question after answering the 5C and dysomeria vaccination intention items.

- Please let us know how you feel about vaccinations. For example, you can explain in a few sentences why you responded the way you did in the previous questions regarding vaccinations.

The following question was asked if participants responded with “Yes” or “No” to the past vaccination behavior item.

- You indicated that you [received/did not receive] the influenza vaccine this past flu season. Please explain the factors that influenced this decision.

### Vaccinations Required & Recommended by University

Required Vaccinations

Illinois requires all college students to be immunized against certain vaccine-preventable diseases. Listed below are **required** vaccinations that are offered at UIUC along with a brief description of the diseases that they prevent. Please take a moment to read and review the immunizations as you answer the questions that follow.

**MMR (Measles, Mumps, Rubella):** starts as simple cold symptoms, but escalates into severe rashes on the face and body, possible permanent hearing loss, damage to the brain and spinal cord, and death.

**T-Dap (Tetanus, Diphtheria, Pertussis):** bacterial infection that can cause painful tightening of the muscles, breathing difficulties, heart failure, paralysis, whooping cough, and possibly death.

| Extremely Unlikely | Unlikely | Neutral | Likely | Extremely Likely |
| --- | --- | --- | --- | --- |
| 1 | 2 | 3 | 4 | 5 |

- I would receive the MMR vaccination even if it was not required to attend [university].
- I would encourage my peers to receive the MMR vaccination even if it was not required by [university].
- I would receive the Tdap vaccination even if it was not required to attend [university].
- I would encourage my peers to receive the Tdap vaccination even if it was not required by [university].

Recommended Vaccinations

[State] recommends all college students to be immunized against certain vaccine-preventable diseases. Listed below are **recommended** vaccinations that are offered at [University] along with a brief description of the diseases that they prevent. Please take a moment to read and review the immunizations as you answer the questions that follow.

**Hepatitis A:**Liver infection that results in symptoms of fatigue, low appetite, stomach pain, nausea, and jaundice, that takes about 2 months to be resolved.

**Meningitis B**: bacterial infection that attacks the brain and spinal cord and cause swelling in those areas. This can be fatal as quickly as 24 hours after the appearance of symptoms.

**Varicella (Chicken Pox):**Highly contagious disease resulting in skin rashes and small, itchy blisters all over the face and body.

| Extremely Unlikely | Unlikely | Neutral | Likely | Extremely Likely |
| --- | --- | --- | --- | --- |
| 1 | 2 | 3 | 4 | 5 |

- I would receive the Hepatitis A vaccination even if it was not recommended by [university].
- I would encourage my peers to receive the Hepatitis A vaccination even if it was not recommended by [university].
- I would receive the Meningitis B vaccination even if it was not recommended by [university].
- I would encourage my peers to receive the Meningitis B vaccination even if it was not recommended by [university].
- I would receive the Varicella vaccination even if it was not recommended by [university].
- I would encourage my peers to receive the Varicella vaccination even if it was not recommended by [university].

### Empathic Concern (Davis, 1980)

For each item, indicate how well it describes you by choosing the appropriate point on the scale. READ EACH ITEM CAREFULLY BEFORE RESPONDING. Answer as honestly as you can.

| 1=Does not describe me well | 2=Only slightly describes me | 3=Describes me moderately well | 4=Describes me  well | 5=Describes me very well |
| --- | --- | --- | --- | --- |

- I often have tender, concerned feelings for people less fortunate than me.
- Sometimes I don't feel very sorry for other people when they are having problems. (reversed)
- When I see someone being taken care of, I feel kind of protective towards them.
- Other people's misfortunes do not usually disturb me a great deal. (reversed)
- When I see someone being treated unfairly, I sometimes don't feel very much pity for them. (reversed)
- I am often quite touched by things that I see happen.
- I would describe myself a pretty soft-hearted person.

### Delayed Gratification (Hoerger et al., 2011)

For each item, indicate how well it describes you by choosing the appropriate point on the scale.

| Strongly Disagree | Disagree | Neither Agree nor Disagree | Agree | Strongly  Agree |
| --- | --- | --- | --- | --- |
| 1 | 2 | 3 | 4 | 5 |

- I would have a hard time sticking with a special, healthy diet. (reversed)
- I have always tried to eat healthy because it pays off in the long term.
- I have given up physical pleasure or comfort to reach my goals.^[[2]](#footnote-2)^
- When faced with a physically demanding chore, I always tried to put off doing it. (reversed)
- I try to consider how my actions will affect other people in the long-term.
- I do not consider how my behavior affects other people. (reversed)
- I try to spend my money wisely.
- I cannot be trusted with money. (reversed)
- I cannot motivate myself to accomplish long-term goals. (reversed)
- I have always felt like my hard work would pay off in the end.

### Risk-Taking (Weber et al., 2002)

For each of the following statements, please indicate the likelihood of you engaging in each activity using the scale provided.

| Extremely Unlikely | Moderately Unlikely | Unsure | Moderately Likely | Extremely Likely |
| --- | --- | --- | --- | --- |
| 1 | 2 | 3 | 4 | 5 |

- Eating 'expired' food products that still 'look okay.'
- Frequent binge drinking.
- Ignoring some persistent physical pain by not going to the doctor.
- Taking a medical drug that has a high likelihood of negative side effects.
- Engaging in unprotected sex.
- Never using sunscreen when you sunbathe.
- Never wearing a seatbelt.
- Not having a smoke alarm in or outside of your bedroom.
- Regularly riding your bicycle without a helmet.
- Smoking/vaping cigarettes every day.

### Behavior Identification Form (Vallacher & Wegner, 1989)

*****PLEASE READ*****

Any behavior can be identified in many ways. For example, one person might describe a behavior as “writing a paper,” while another person might describe the behavior as “pushing keys.” Yet another person might describe the behavior as “expressing thoughts.” We are interested in your personal preferences for how a number of different behaviors should be described. On the following pages you will find several different behaviors listed. After each behavior will be two choices of different ways in which the behavior might be identified. For example: “Attending class” might be best described as “sitting in a chair” or “looking at a teacher.”

For the next segment of the study, your task is to choose the identification that best describes each behavior for you. That is, select the description that you personally believe is more appropriate from each pair. There is no right or wrong answer, we are interested in your personal preference.

- Making a list [Getting organized* / Writing things down]
- Reading [Following lines of print / Gaining knowledge*]
- Joining the Army [Helping the Nation’s defense* / Signing up]
- Washing Clothes [Removing odors from clothes* / Putting clothes into the machine]
- Picking an apple [Getting something to eat* / Pulling an apple off a branch]
- Chopping down a tree [Wielding an axe / Getting firewood*]
- Measuring a room for carpeting [Getting ready to remodel* / Using a yardstick]
- Cleaning the house [Showing one’s cleanliness* / Vacuuming the floor]
- Painting a room [Applying brush strokes / Making the room look fresh*]
- Paying the rent [Maintaining a place to live* / Writing a check]
- Caring for houseplants [Watering plants / Making the room look nice*]
- Locking the door [Putting a key in the lock / Securing the house*]
- Voting [Influencing the election* / Marking a ballot]
- Climbing a tree [Getting a good view* / Holding on to branches]
- Filling out a personality test [Answering questions / Revealing what you’re like*]
- Toothbrushing [Preventing tooth decay* / Moving a brush around in one’s mouth]
- Taking a test [Answering questions / Showing one’s knowledge*]
- Greeting someone [Saying hello / Showing friendliness*]
- Resisting temptation [Saying “no” / Showing moral courage*]
- Eating [Getting nutrition* / Chewing and swallowing]
- Growing a garden [Planting seeds / Getting fresh vegetables*]
- Traveling by a car [Following a map / Seeing countryside*]
- Having a cavity filled [Protecting your teeth* / Going to the dentist]
- Talking to a child [Teaching the child something* / Using simple words]
- Pushing a doorbell [Moving a finger / Seeing if someone’s home*]

## Supplementary Measures: Study 2 (Online Panel Workers)

### Open-Ended Questions

Participants responded to the following question after answering the COVID-19/dysomeria vaccination intention questions.

- Please let us know how you feel about vaccinations. For example, you can explain in a few sentences why you responded the way you did in the previous questions regarding vaccinations.

### COVID-19 Optimism Bias

In the following questions we will ask you to answer questions for you and an average person similar to you. By "average person similar to you" we mean someone of the same gender and ethnicity, roughly your age, who lives in the same town/city/area. When we say average person in the following, please think of this person.

|  | **(0%) Definitely not** | | **Definitely (100%)** | |
| --- | --- | --- | --- | --- |
|  | | 0 | | 100 |

| What do you think is the probability that an average person will be infected with the  novel coronavirus in the next 2 months? | 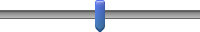 |
| --- | --- |
| If an average person were to be infected with the new coronavirus, how probable would it be that they get only mild symptoms like a common cold? | 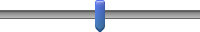 |
| What do you think is the probability that you will be infected with the novel coronavirus in  the next 2 months? | 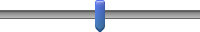 |
| If you were to be infected with the new coronavirus, how probable would it be that you get only mild symptoms like a common cold? | 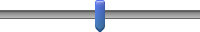 |

### Reasons Influencing COVID-19 Vaccination

In the earlier question asking about the COVID-19 vaccine, you indicated: [“I would not receive the vaccination even if it’s free ($0)” / “I would receive the vaccination only if it’s free ($0); If I need to pay money, I would not receive the vaccination” / “I would pay money to receive the vaccination”]. Listed below are some concerns that people may have about the vaccine.

1. Please rate your agreement with each statement.
2. Then, indicate whether each statement matters to your decision about getting COVID-19 vaccination.
3. If you did not initially think about the reason when you answered the question earlier, please let us know by checking the box on the right.

| Please indicate your agreement with each statement. | | | | | | | Does this reason matter for your decision to vaccinate? | |
| --- | --- | --- | --- | --- | --- | --- | --- | --- |
| Strongly Disagree | Disagree | Somewhat Disagree | Neutral | Somewhat Agree | Agree | Strongly Agree | No | Yes |
| 1 | 2 | 3 | 4 | 5 | 6 | 7 |  |  |

- The vaccine is too new.
- I worry about the side effects.
- The vaccine will not protect me.
- I avoid most vaccines.
- COVID-19 is not severe enough to concern me.
- A doctor has recommended no vaccines.
- I will not have access to the vaccine.
- My religion prevents vaccination.

### Attitudes about Social Distancing

To what extent do you believe it is important to behave in the following ways?

| 1=Not at all important | 2 | 3 | 4 | 5 | 6 | 7=Extremely important |
| --- | --- | --- | --- | --- | --- | --- |

- Avoid visiting an elderly relative.
- Keep 6 feet of distance between yourself and other people while in an enclosed area (e.g., in line at a store).
- Wear a mask while in an enclosed area with others (e.g., in a store).
- Avoid hosting an indoor get-together with friends.
- Avoid visiting non-essential businesses (e.g., nail salon, hair stylist, bowling alley).
- Avoid travel to other locations (e.g., out of state) to visit friends or family.

### Perceived Health/Economic Impact of COVID-19

| 1=Not at all bad/serious | 2 | 3 | 4 | 5 | 6 | 7=Extremely bad/serious |
| --- | --- | --- | --- | --- | --- | --- |

- To what extent, in your view, has COVID-19 had a negative impact on health in the U.S? The impacts of COVID-19 on U.S. health have been...
- To what extent, in your view, has COVID-19 had a negative impact on economy in the U.S? The impacts of COVID-19 on U.S. economy have been...

In your opinion, which is more important: protecting public health in the U.S. or trying to revive the U.S. economy?

| 1=Protecting public health is more important | 2 | 3 | 4=Both are equally important | 5 | 6 | 7=Reviving the U.S. economy is more important |
| --- | --- | --- | --- | --- | --- | --- |

### COVID-19 Media Consumption

There are many resources available to stay informed on COVID-19. Which sources are you using to receive Coronavirus-related information? Please select all that apply. [ABC News / BBC / CBS News / CDC (Centers for Disease Control and Prevention) / CNN / FOX News / MSNBC / NBC News / NPR / PBS / Social Media (e.g., Twitter, Facebook, Instagram, TikTok) / State-government websites / The Guardian / The New York Times / The Rush Limbaugh Show (radio) / The Sean Hannity Show (radio) / The Wall Street Journal / The Washington Post / Univision / USA Today / WHO (World Health Organization) / Other (please specify)]

# SUPPLEMENTARY RESULTS AND ANALYSES

Here, we report additional results and analyses that provide converging evidence and serve to supplement the primary findings presented in the main text. Below, we present descriptive statistics of supplementary measures, and additional models predicting vaccination intention measures including pre-registered analyses.

## Study 1 Supplementary Analyses

### Attention Check

There were two attention checks in Study 1. The first item asked: If you’re reading this, please check “Strongly Agree.” Out of 346 respondents, n = 34 failed this question and selected a response other than “Strongly Agree.” The second item was displayed at the end of the survey and asked whether they took the study seriously and responded thoughtfully. Out of 346 respondents, n = 10 responded that they did not take the study seriously. In summary, 28 respondents failed the first item only, 4 respondents failed the second item only, and 6 respondents failed both items, leaving us with the final sample size of *N* = 346 - 28 - 4 - 6 = 308 consisting of participants who passed both attention check items.

### Supplementary Variables Descriptive Statistics

Table S2: Descriptive statistics for supplementary measures and correlations with COVID-19 and dysomeria vaccination intentions in Study 1.

|  |  |  |  | **COVID vaccine** | | **Dysomeria vaccine** | |
| --- | --- | --- | --- | --- | --- | --- | --- |
| **Variable** | ***α*** | **M** | **SD** | **r** | **p** | **r** | **p** |
| Receiving Vaccines | .95 | 4.54 | 0.73 | .39 | <.001 | .52 | <.001 |
| Encouraging Peers | .97 | 4.23 | 0.96 | .41 | <.001 | .40 | <.001 |
| Empathic Concern | .79 | 4.00 | 0.64 | .02 | .704 | .05 | .393 |
| Delaying Gratification | .70 | 3.78 | 0.50 | -.02 | .776 | -.02 | .774 |
| Risk-Taking | .61 | 2.27 | 0.57 | -.03 | .584 | .09 | .107 |
| BIF | .85 | 13.15 | 5.58 | -.06 | .313 | -.03 | .601 |

Note. BIF = Behavior Identification Form. The composite score for delaying gratification is the sum of all 25 items with greater numbers indicating higher abstraction.

### Main Variables Correlations

Table S3: Study 1 (undergraduates) means, standard deviations, and correlations of measures for the full sample (left side/below diagonal) and vaccine-hesitant subset (right side/above diagonal).

| Variables | *M* | *SD* | 1 | 2 | 3 | 4 | 5 | 6 | 7 | 8 | 9 | 10 | 11 | 12 | 13 | 14 | 15 | *M* | *SD* |
| --- | --- | --- | --- | --- | --- | --- | --- | --- | --- | --- | --- | --- | --- | --- | --- | --- | --- | --- | --- |
| 01.COVID | 5.10 | 1.83 | - | **.39*** | **.45*** | **.40*** | **-.27** | -.08 | **-.28** | .16 | **-.42*** | **-.36*** | **-.32*** | -.03 | .08 | .01 | -.22† | 4.58 | 2.03 |
| 02.Dysomeria | 6.36 | 1.09 | **.31*** | - | **.54*** | **.62*** | **-.48*** | **-.29*** | **-.37*** | .16 | **-.42*** | **-.50*** | **-.30*** | .08 | .14 | -.10 | **-.30*** | 6.14 | 1.36 |
| 03.Confidence | 5.74 | 0.95 | **.41*** | **.42*** | .61 | **.59*** | **-.47*** | **-.24** | **-.39*** | **.34*** | **-.59*** | **-.73*** | **-.53*** | .12 | .11 | -.02 | **-.23** | 5.47 | 1.05 |
| 04.Collective | 6.42 | 0.93 | **.38*** | **.49*** | **.52*** | .44 | **-.58*** | **-.34*** | **-.32*** | .16 | **-.55*** | **-.52*** | **-.35*** | .01 | .14 | .04 | **-.24** | 6.21 | 1.13 |
| 05.Complacent | 2.04 | 1.05 | **-.23*** | **-.37*** | **-.34*** | **-.51*** | .52 | **.31*** | **.25** | -.12 | **.41*** | **.49*** | **.35*** | .04 | **-.26** | -.03 | **.41*** | 2.23 | 1.17 |
| 06.Constraint | 1.94 | 1.08 | **-.19*** | **-.27*** | **-.27*** | **-.40*** | **.37*** | .69 | .17† | **-.27** | **.25** | **.31*** | **.23** | -.17 | .03 | .02 | .14 | 2.26 | 1.15 |
| 07.Calculation | 3.86 | 1.66 | **-.20*** | **-.29*** | **-.23*** | **-.25*** | **.25*** | **.18** | .78 | .04 | **.26** | **.34*** | .20† | .06 | .00 | .18† | **.23** | 4.15 | 1.71 |
| 08.Knowledge | 4.97 | 1.17 | **.22*** | **.19*** | **.37*** | **.23*** | **-.18** | **-.29*** | .03 | .88 | **-.31*** | **-.34*** | **-.39*** | **.33*** | .00 | .15 | -.03 | 4.64 | 1.26 |
| 09.Conspiracy | 1.95 | 0.90 | **-.32*** | **-.34*** | **-.50*** | **-.50*** | **.35*** | **.27*** | **.18** | **-.33*** | .83 | **.73*** | **.58*** | -.17† | -.01 | .02 | .14 | 2.17 | 1.05 |
| 10.Danger | 2.78 | 1.18 | **-.33*** | **-.46*** | **-.60*** | **-.47*** | **.36*** | **.33*** | **.25*** | **-.40*** | **.70*** | .90 | **.60*** | -.16 | -.09 | .00 | **.24** | 3.20 | 1.26 |
| 11.Mistrust | 1.77 | 0.64 | **-.27*** | **-.24*** | **-.45*** | **-.36*** | **.30*** | **.26*** | .13† | **-.36*** | **.53*** | **.56*** | .86 | **-.28** | -.05 | -.12 | .18† | 1.86 | 0.67 |
| 12.Analytic | 3.78 | 0.58 | .08 | **.17** | **.19*** | .10 | -.03 | **-.18** | -.01 | **.35*** | **-.21*** | **-.25*** | **-.30*** | .83 | -.04 | .12 | .06 | 3.72 | 0.62 |
| 13.Vulnerable | 3.73 | 1.17 | .05 | .06 | .04 | .11 | **-.27*** | -.02 | .01 | .03 | .01 | .02 | -.02 | -.06 | .88 | -.01 | -.20† | 3.73 | 1.26 |
| 14.IOS | 4.28 | 1.56 | .09 | -.08 | -.01 | .06 | -.09 | -.06 | .07 | .12† | -.02 | -.03 | -.10 | .09 | .03 | - | .14 | 4.14 | 1.62 |
| 15.Conservative | 3.18 | 1.49 | -.05 | **-.21*** | **-.21*** | **-.17** | **.36*** | .14† | **.16** | -.02 | .10 | **.18** | .12† | -.06 | -**.15** | .07 | - | 3.26 | 1.41 |
| 16.Past behavior | 0.57 | 0.50 | **.25*** | **.18** | **.25*** | **.20*** | **-.15** | **-.26*** | **-.15** | **.24*** | **-.21*** | **-.31*** | -.13† | .08 | .01 | .08 | -.05 | - | - |

Below the diagonal are correlations for the full sample (*N* = 308). Above the diagonal are correlations for participants who did not report receiving the flu shot during 2019-2020 flu season (*N* = 133). Respectively, *M* and *SD* on the left and right are for the full and reduced samples. On the diagonal are *α*/*r*, computed using the full sample. COVID = COVID-19 vaccination intention, Dysomeria = hypothetical child vaccination intention, Confidence to Calculation = subscales of the 5C Antecedents of Vaccination, Knowledge = self-reported vaccine knowledge, Conspiracy = anti-vaccine conspiracy beliefs, Danger = perceived danger of vaccines, Mistrust = mistrust in science/scientists, Analytic = analytical thinking style, Vulnerable = perceived disease vulnerability, IOS = inclusion of other (acquaintances) in the self, Conservative = ideological conservatism, Past behavior = dummy variable where receiving the seasonal flu shot was coded as 1. † *p* < .05, bolded *p* < .01, * *p* < .001

### Robustness Check for Time

In Model 1b of Table 2 in the main text, we controlled for COVID-19 U.S. deaths. Below, we present the same models controlling for COVID-19 U.S. cases (Table S4 Model 1X), and whether respondents participated before or after Spring Break when the university fully transitioned to remote-learning (Table S4 Model 2X). These additional models revealed virtually identical results as the model presented in the main text.

Table S4: OLS regression models predicting COVID-19 vaccination intentions in Study 1.

|  | **Model 1X** | | **Model 2X** | |
| --- | --- | --- | --- | --- |
| **Predictor** | **β** | **95% CI** | **β** | **95% CI** |
|  |  |  |  |  |
| 5C: Confidence | .23** | [.09, .37] | .22** | [.08, .36] |
| 5C: Collective | .18* | [.04, .32] | .18** | [.04, .32] |
| 5C: Complacency | .01 | [-.12, .15] | .01 | [-.12, .15] |
| 5C: Constraint | .02 | [-.09, .14] | .02 | [-.10, .14] |
| 5C: Calculation | -.10† | [-.21, .02] | -.10† | [-.21, .02] |
| Vaccine Knowledge | .06 | [-.06, .18] | .06 | [-.06, .18] |
| Conspiracy Belief | -.08 | [-.23, .07] | -.08 | [-.23, .07] |
| Vaccine Danger | .01 | [-.15, .18] | .01 | [-.15, .18] |
| Science Mistrust | -.05 | [-.18, .08] | -.05 | [-.18, .08] |
| Analytic Thinking | -.02 | [-.14, .09] | -.02 | [-.14, .09] |
| Disease Vulnerability | .03 | [-.08, .14] | .03 | [-.08, .14] |
| Self-Other Overlap | .08 | [-.02, .19] | .08 | [-.02, .19] |
| Conservatism | .06 | [-.05, .18] | .06 | [-.05, .18] |
| Past Behavior (1=flu shot received) | .23* | [.01, .45] | .23* | [.01, .45] |
| Gender (1=female) | .07 | [-.19, .33] | .07 | [-.19, .33] |
| Ethnicity (1=non-White) | .14 | [-.08, .37] | .15 | [-.08, .37] |
| COVID US cases (log) | -.05 | [-.16, .05] |  |  |
| Time (1=post-break) |  |  | -.13 | [-.37, .12] |
|  |  |  |  |  |
| *N* | 307 | | 307 | |
| R^2^ /Adjusted R^2^ | .25/.21 | | .25/.21 | |

*Note.* Coefficients are standardized. † *p* < .10, * *p* < .05, ** *p* < .01

## Study 2 Supplementary Analyses

### Attention Check

For Study 2, we had three pre-registered exclusion criteria: respondents who 1) do not finish the survey, 2) fail to answer one or more attention checks correctly, and 3) fail to spend more than 10 seconds on pages involving reading and/or responding to multiple items. We received 848 complete responses in Study 2. The first attention check item was identical to Study 1. Out of 848 respondents, n = 117 failed to select “Strongly Agree.” The second attention check item asked respondents to select “67” on a sliding scale. Out of 848 respondents, n = 109 provided a response other than 67. In summary, 58 respondents failed the first item only, 50 respondents failed the second item only, and 59 respondents failed both items, leaving us with n = 848 - 58 - 50 - 59 = 681 respondents who passed both attention check items. Finally, we checked the average reading time and excluded n = 5 respondents who spent less than 10 seconds per page. Thus, the final sample size for Study 2 is *N* = 681 - 5 = 676.

### Additional Demographics

Table S5:Participants’ state of residence and COVID-19 cases and deaths (Study 2).

| **State** | **Number of**  **Participants** | **Governor’s**  **Affiliation** | **COVID-19**  **Cases** | **COVID-19**  **Deaths** |
| --- | --- | --- | --- | --- |
| Alabama | 17 | Republican | 67,011 | 1,287 |
| Alaska | 1 | Republican | 1,874 | 18 |
| Arizona | 16 | Republican | 143,624 | 2,761 |
| Arkansas | 4 | Republican | 33228 | 357 |
| California | 68 | Democrat | 384,692 | 7,685 |
| Colorado | 14 | Democrat | 40,142 | 1,752 |
| Connecticut | 8 | Democrat | 47,893 | 4,396 |
| Delaware | 3 | Democrat | 13,519 | 523 |
| Florida | 58 | Republican | 345,612 | 4,982 |
| Georgia | 25 | Republican | 143,123 | 3,173 |
| Hawaii | 4 | Democrat | 1,273 | 24 |
| Idaho | 2 | Republican | 14,873 | 119 |
| Illinois | 17 | Democrat | 162,750 | 7,488 |
| Indiana | 17 | Republican | 56,571 | 2,822 |
| Iowa | 8 | Republican | 38,723 | 793 |
| Kansas | 3 | Democrat | 21,965 | 299 |
| Kentucky | 5 | Democrat | 23,161 | 670 |
| Louisiana | 6 | Democrat | 91,706 | 3,543 |
| Maine | 2 | Democrat | 3,711 | 117 |
| Maryland | 18 | Republican | 78,685 | 3,382 |
| Massachusetts | 8 | Republican | 113,534 | 8,431 |
| Michigan | 9 | Democrat | 81,868 | 6,366 |
| Minnesota | 10 | Democrat | 46,204 | 1,581 |
| Mississippi | 3 | Republican | 43,889 | 1,358 |
| Missouri | 15 | Republican | 33,094 | 1,129 |
| Montana | 5 | Democrat | 2,533 | 37 |
| Nebraska | 1 | Republican | 22,583 | 301 |
| Nevada | 12 | Democrat | 35,977 | 673 |
| New Hampshire | 3 | Republican | 6,203 | 398 |
| New Jersey | 19 | Democrat | 176,783 | 15,706 |
| New Mexico | 3 | Democrat | 16,971 | 571 |
| New York | 64 | Democrat | 408,495 | 32,198 |
| North Carolina | 20 | Democrat | 99,778 | 1,634 |
| North Dakota | 3 | Republican | 5,126 | 93 |
| Ohio | 26 | Republican | 74,932 | 3,174 |
| Oklahoma | 6 | Republican | 25,265 | 451 |
| Oregon | 14 | Democrat | 14,579 | 260 |
| Pennsylvania | 34 | Democrat | 101,738 | 7,018 |
| Rhode Island | 2 | Democrat | 17,793 | 990 |
| South Carolina | 12 | Republican | 69,986 | 1,155 |
| South Dakota | 1 | Republican | 7,906 | 118 |
| Tennessee | 15 | Republican | 78,115 | 843 |
| Texas | 46 | Republican | 325,030 | 3,958 |
| Utah | 5 | Republican | 34,682 | 243 |
| Vermont | 1 | Republican | 1,350 | 56 |
| Virginia | 12 | Democrat | 78,375 | 2,031 |
| Washington | 14 | Democrat | 46,946 | 1,447 |
| West Virginia | 3 | Republican | 5,042 | 100 |
| Wisconsin | 13 | Democrat | 45,948 | 851 |
| Wyoming | 1 | Republican | 2,126 | 24 |

### Main Variables Correlations

Table S6: Study 2 (online panel workers) means, standard deviations, and correlations of measures for the full sample (left side/below diagonal) and vaccine-hesitant subset (right side/above diagonal).

| Variables | *M* | *SD* | 1 | 2 | 3 | 4 | 5 | 6 | 7 | 8 | 9 | 10 | 11 | 12 | 13 | 14 | 15 | *M* | *SD* |
| --- | --- | --- | --- | --- | --- | --- | --- | --- | --- | --- | --- | --- | --- | --- | --- | --- | --- | --- | --- |
| 01.COVID | 4.57 | 1.96 | **-** | **.50*** | **.56*** | **.43*** | **-.27*** | .02 | -.10 | .13† | **-.34*** | **-.38*** | **-.33*** | .05 | **.27*** | **.15** | **-.25*** | 3.92 | 2.13 |
| 02.Dysomeria | 5.65 | 1.82 | **.45*** | **-** | **.61*** | **.59*** | **-.46*** | **-.19** | .00 | .12† | **-.48*** | **-.47*** | **-.44*** | .03 | **.19*** | .06 | **-.25*** | 5.10 | 2.13 |
| 03.Confidence | 5.05 | 1.45 | **.50*** | **.56*** | .73 | **.64*** | **-.42*** | -.03 | -.06 | .14† | **-.53*** | **-.61*** | **-.57*** | .01 | **.17** | **.23*** | **-.32*** | 4.52 | 1.56 |
| 04.Collective | 5.73 | 1.34 | **.36*** | **.51*** | **.59*** | .71 | **-.61*** | **-.23*** | -.01 | .01 | **-.60*** | **-.57*** | **-.54*** | .10 | **.17** | .05 | **-.29*** | 5.11 | 1.43 |
| 05.Complacent | 2.39 | 1.51 | **-.22*** | **-.42*** | **-.37*** | **-.64*** | .82 | **.35*** | .09 | **.17** | **.66*** | **.58*** | **.53*** | .00 | **-.29*** | .11 | **.27*** | 3.02 | 1.54 |
| 06.Constraint | 2.33 | 1.49 | -.06 | **-.22*** | **-.16*** | **-.40*** | **.54*** | .83 | -.08 | -.14† | **.32*** | **.32*** | **.22*** | **-.20*** | .14† | -.02 | -.07 | 2.75 | 1.48 |
| 07.Calculation | 5.30 | 1.38 | -.07 | -.03 | -.03 | -.02 | **.10** | .06 | .77 | .13† | .09 | **.19*** | .12† | .06 | -.10 | .04 | .08 | 5.37 | 1.31 |
| 08.Knowledge | 4.93 | 1.37 | **.21*** | **.23*** | **.29*** | **.18*** | -.01 | **-.16*** | **.14*** | .92 | .01 | -.07 | -.02 | **.39*** | -.15† | **.22*** | .04 | 4.57 | 1.46 |
| 09.Conspiracy | 2.57 | 1.43 | **-.27*** | **-.45*** | **-.47*** | **-.62*** | **.70*** | **.54*** | **.13*** | -.09† | .91 | **.78*** | **.67*** | -.09 | -.09 | -.02 | **.28*** | 3.01 | 1.49 |
| 10.Danger | 3.39 | 1.44 | **-.35*** | **-.45*** | **-.55*** | **-.59*** | **.60*** | **.50*** | **.23*** | **-.21*** | **.78*** | .92 | **.69*** | -.11† | -.11 | -.06 | **.29*** | 3.93 | 1.41 |
| 11.Mistrust | 2.22 | 1.05 | **-.33*** | **-.42*** | **-.49*** | **-.55*** | **.56*** | **.40*** | **.15*** | **-.14*** | **.68*** | **.67*** | .94 | **-.16** | -.08 | .01 | **.45*** | 2.51 | 1.09 |
| 12.Analytic | 3.67 | 0.77 | **.11** | .10† | **.11** | **.17*** | **-.12** | **-.25*** | .04 | **.37*** | **-.19*** | **-.23*** | **-.26*** | .88 | **-.25*** | .07 | .02 | 3.62 | 0.78 |
| 13.Vulnerable | 3.55 | 1.19 | **.16*** | **.14*** | **.11** | **.15*** | **-.22*** | .06 | -.07 | -.04 | -.04 | -.03 | -.05 | **-.15*** | .84 | -.14† | -**.26*** | 3.33 | 1.20 |
| 14.IOS | 3.47 | 1.91 | **.19*** | .09† | **.23*** | **.11** | .00 | -.09† | .08† | **.25*** | -.05 | -.10† | -.09† | **.17*** | **-.11** | **-** | .08 | 3.19 | 1.93 |
| 15.Conservative | 4.03 | 1.89 | **-.24*** | **-.24*** | **-.28*** | **-.25*** | **.22*** | .05 | **.10** | -.04 | **.25*** | **.29*** | **.45*** | **-.10** | **-.16*** | .02 | **-** | 4.18 | 1.86 |
| 16.Past behavior | 0.56 | 0.50 | **.29*** | **.27*** | **.33*** | **.41*** | **-.37*** | **-.25*** | -.04 | **.23*** | **-.27*** | **-.33*** | **-.25*** | .06 | **.16*** | **.13*** | -.07 | - | - |

Below the diagonal are correlations for the full sample (*N* = 676). Above the diagonal are correlations for participants who reported not receiving the flu shot during 2019-2020 flu season (*N* = 299). Respectively, *M* and *SD* on the left and right are for the full and reduced samples. On the diagonal are *α* computed using the full sample. COVID = COVID-19 vaccination intention, Dysomeria = hypothetical child vaccination intention, Confidence to Calculation = subscales of the 5C Antecedents of Vaccination, Knowledge = self-reported vaccine knowledge, Conspiracy = anti-vaccine conspiracy beliefs, Danger = perceived danger of vaccines, Mistrust = mistrust in science/scientists, Analytic = analytical thinking style, Vulnerable = perceived disease vulnerability, IOS = inclusion of other (community members) in the self, Conservative = ideological conservatism, Past behavior = dummy variable where receiving the seasonal flu shot was coded as 1. † *p* < .05, bolded *p* < .01, * *p* < .001

### Robustness Check with COVID meta-data

In Model 1b of Table 4 in the main text, we controlled for COVID-19 state deaths. Below, we present the same models controlling for COVID-19 state cases (Table S7 Model 3X) and for political party affiliation of the governor of state of residence (Table S7 Model 4X). These additional models revealed virtually identical results as the model presented in the main text.

Table S7: OLS regression models predicting COVID-19 vaccination intentions in Study 2.

|  | **Model 3X** | | **Model 4X** | |
| --- | --- | --- | --- | --- |
| **Predictor** | **β** | **95% CI** | **β** | **95% CI** |
|  |  |  |  |  |
| 5C: Confidence | .27*** | [.18, .35] | .27*** | [.18, .35] |
| 5C: Collective | .09† | [.00, .19] | .09† | [.00, .19] |
| 5C: Complacency | .03 | [-.07, .13] | .03 | [-.07, .13] |
| 5C: Constraint | .06 | [-.03, .14] | .06 | [-.02, .14] |
| 5C: Calculation | -.03 | [-.10, .03] | -.03 | [-.10, .03] |
| Vaccine Knowledge | .00 | [-.07, .07] | .00 | [-.07, .07] |
| Conspiracy Belief | .00 | [-.11, .12] | .00 | [-.11, .12] |
| Vaccine Danger | -.04 | [-.15, .07] | -.04 | [-.15, .07] |
| Science Mistrust | -.08 | [-.18, .02] | -.08 | [-.17, .02] |
| Analytic Thinking | .00 | [-.07, .07] | .00 | [-.07, .07] |
| Disease Vulnerability | .12*** | [.06, .19] | .12*** | [.06, .19] |
| Self-Other Overlap | .07* | [.00, .13] | .07* | [.00, .13] |
| Conservatism | -.10** | [-.17, -.03] | -.10** | [-.17, -.03] |
| Past Behavior (1=flu shot received) | .24*** | [.10, .38] | .24*** | [.10, .38] |
| Gender (1=female) | -.21** | [-.34, -.08] | -.21** | [-.35, -.08] |
| Ethnicity (1=non-White) | .00 | [-.16, .16] | .00 | [-.16, .16] |
| Age | -.02 | [-.10, .05] | -.02 | [-.10, .05] |
| Income | .26*** | [.19, .32] | .26*** | [.19, .32] |
| COVID state cases (log) | -.01 | [-.07, .05] |  |  |
| COVID state deaths (log) |  |  | -.01 | [-.07, .06] |
| Governor (1=Republican) |  |  | .00 | [-.12, .13] |
|  |  |  |  |  |
| *N* | 670 | | 670 | |
| R^2^ /Adjusted R^2^ | .40/.38 | | .40/.38 | |

*Note.* Coefficients are standardized. † *p* < .10, * *p* < .05, ** *p* < .01, *** *p* < .001

### Optimism Bias Regarding COVID-19 Infection and Symptoms

As stated in our pre-registration, we computed difference scores for the optimism bias items such that positive difference scores indicated greater optimism/self-serving bias. Specifically, we computed the following:

- Infection optimism bias = [Perceived likelihood (%) of an average person being infected] – [Perceived likelihood (%) of self being infected]
- Symptom optimism bias = [Perceived likelihood (%) of self having minor symptoms)] – [Perceived likelihood (%) of an average person having minor symptoms]

Table S8 shows the means, standard deviations, and correlations with vaccination intentions for the original optimism score items and the optimism score difference scores.

Table S8: Descriptive statistics for optimism bias and correlations with COVID-19 and dysomeria vaccination intentions in Study 2.

|  |  |  | **COVID vaccine** | | **Dysomeria vaccine** | |
| --- | --- | --- | --- | --- | --- | --- |
| **Variable** | **M** | **SD** | **r** | **p** | **r** | **p** |
| Infection (Other) | 52.09 | 27.94 | .21 | <.001 | .22 | <.001 |
| Infection (Self) | 37.20 | 26.56 | .25 | <.001 | .18 | <.001 |
| Mild Symptoms (Other) | 60.81 | 23.03 | -.08 | .048 | -.07 | .091 |
| Mild Symptoms (Self) | 53.68 | 27.43 | -.11 | .004 | -.14 | <.001 |
| Infection Optimism Bias | 14.90 | 22.79 | -.03 | .372 | .05 | .179 |
| Symptoms Optimism Bias | -7.15 | 23.83 | -.05 | .161 | -.10 | .008 |

A paired *t*-test revealed that participants believed that the probability of infection was greater for an average person than themselves, *t*(675) = 17.00, *p* < .001, CI_.95_ = [13.18, 16.62]. However, participants believed that if they were to be infected with COVID, they would be less likely to get only mild symptoms relative to an average person, *t*(674) = 7.79, *p* < .001, CI_.95_ = [5.35, 8.95].

### COVID-19 Vaccination Intention (Continuous Measure)

As stated in our pre-registration, we estimated an OLS regression model predicting COVID-19 vaccination intentions including the optimism bias difference scores. Results are shown in Table S9. Here, coefficients are standardized, and the outcome variable in Model 5X (COVID-19 vaccination intention) is the amount of money participants indicated on the slider.

Table S9: OLS regression model (Study 2) predicting COVID-19 vaccination intentions (continuous measure).

|  | **Model 5X** | | |
| --- | --- | --- | --- |
| **Predictor** | **β** | **95% CI** | **Semi-partial *r*** |
|  |  |  |  |
| 5C: Confidence | .16** | [.06, .26] | .11 |
| 5C: Collective | .07 | [-.04, .18] | .04 |
| 5C: Complacency | .09 | [-.03, .20] | .05 |
| 5C: Constraint | .14** | [.05, .24] | .11 |
| 5C: Calculation | .02 | [-.05, .10] | .02 |
| Vaccine Knowledge | .11* | [.02, .19] | .09 |
| Conspiracy Belief | .13† | [-.01, .27] | .07 |
| Vaccine Danger | -.14* | [-.27, .00] | -.07 |
| Science Mistrust | .00 | [-.11, .11] | .00 |
| Analytic Thinking | .03 | [-.05, .12] | .03 |
| Disease Vulnerability | .07† | [-.01, .15] | .06 |
| Self-Other Overlap | .12** | [.04, .20] | .11 |
| Conservatism | -.08† | [-.16, .00] | -.07 |
| Past Behavior (1=flu shot received) | .10 | [-.06, .27] | .04 |
| Infection Optimism Bias | .01 | [-.06, .09] | .01 |
| Symptom Optimism Bias | .01 | [-.07, .08] | .01 |
|  |  |  |  |
| *N* | 673 | | |
| R^2^ /Adjusted R^2^ | .15/.13 | | |

*Note.* Coefficients are standardized. † *p* < .10, * *p* < .05, ** *p* < .01

Figure S1: The distribution of responses to “I would pay up to $__ for the vaccine” in Study 2.


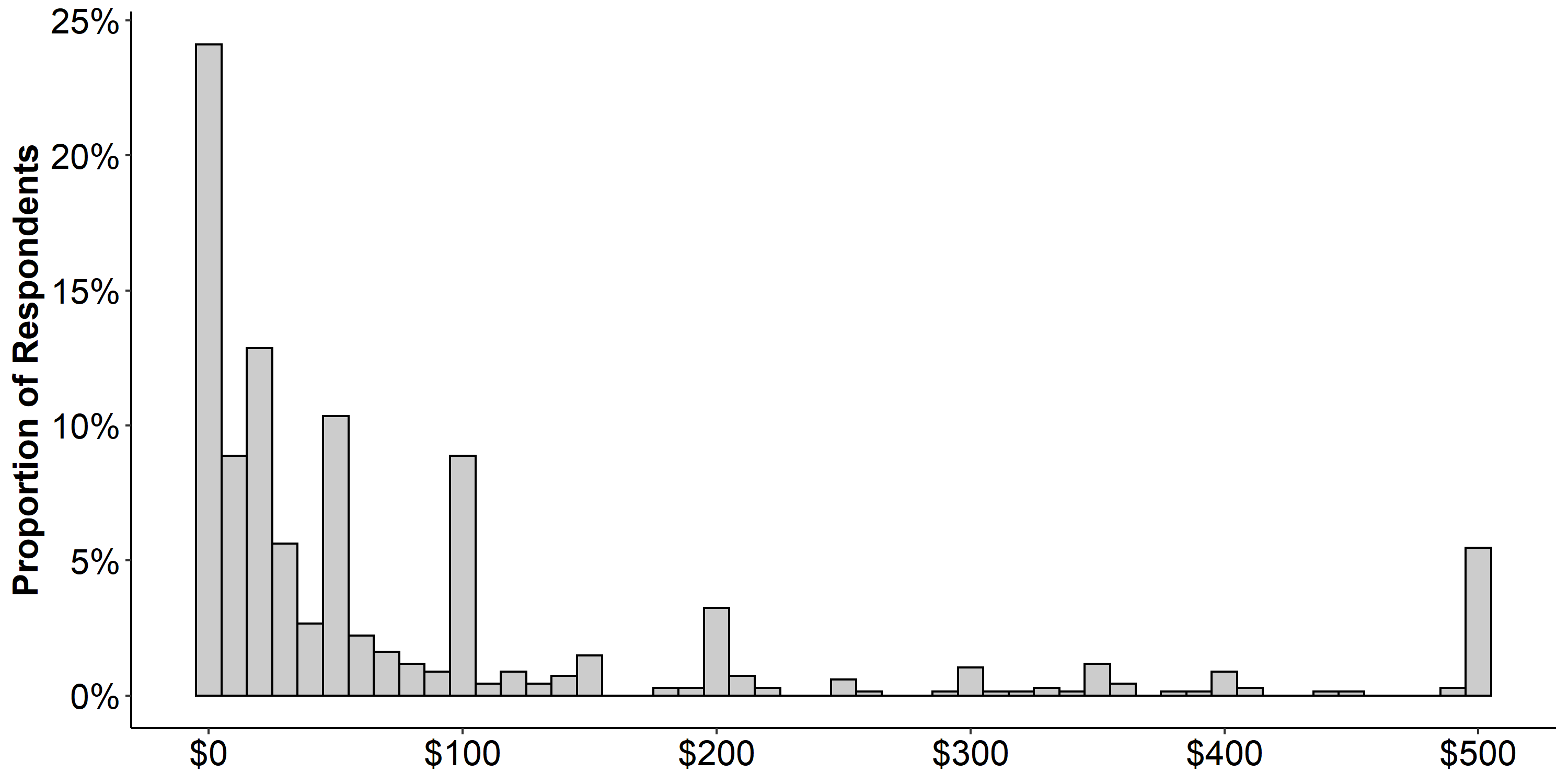


Because of the zero-inflated pattern of the original COVID-19 vaccination intention measure (see Figure S1), we also estimated the zero-inflated negative binomial (ZINB) model with the same predictors. A ZINB model simultaneously predicts two things: grouping (probability of being in the group paying $0) and count (amount of money willing to pay if not in the $0 group). All continuous measures were mean-centered in this model.

Table S10: ZINB model predicting COVID-19 vaccination intention (continuous measure) in Study 2.

| **Logit Model 6X** | **b** | **p** | **Odds Ratio** | **95% CI** |
| --- | --- | --- | --- | --- |
| Intercept | -2.38 | <.001 |  |  |
| 5C: Confidence | -0.54 | <.001 | 0.58 | [0.44, 0.77] |
| 5C: Collective | -0.15 | .360 | 0.86 | [0.62, 1.19] |
| 5C: Complacency | -0.11 | .484 | 0.90 | [0.67, 1.21] |
| 5C: Constraint | 0.04 | .735 | 1.04 | [0.82, 1.33] |
| 5C: Calculation | 0.26 | .081 | 1.30 | [0.97, 1.74] |
| Vaccine Knowledge | -0.03 | .825 | 0.97 | [0.76, 1.24] |
| Conspiracy Belief | 0.33 | .077 | 1.40 | [0.96, 2.02] |
| Vaccine Danger | 0.02 | .931 | 1.02 | [0.68, 1.53] |
| Science Mistrust | 0.25 | .268 | 1.28 | [0.83, 1.99] |
| Analytic Thinking | -0.01 | .953 | 0.99 | [0.63, 1.54] |
| Disease Vulnerability | -0.41 | .008 | 0.66 | [0.49, 0.90] |
| Self-Other Overlap | -0.05 | .596 | 0.95 | [0.80, 1.13] |
| Conservatism | 0.11 | .294 | 1.12 | [0.91, 1.38] |
| Past Behavior (1=flu shot received) | -1.20 | .001 | 0.30 | [0.14, 0.62] |
| Gender (1=female) | 0.41 | .235 | 1.51 | [0.76, 2.99] |
| Ethnicity (1=non-White) | -0.18 | .653 | 0.83 | [0.37, 1.85] |
| Age | -0.01 | .197 | 0.99 | [0.97, 1.01] |
| Income | -0.37 | .002 | 0.69 | [0.55, 0.87] |
|  |  |  |  |  |
| **Count Model 6X** | **b** | **p** | **IRR** | **95% CI** |
| Intercept | 4.53 | <.001 |  |  |
| 5C: Confidence | 0.14 | .003 | 1.15 | [1.05, 1.26] |
| 5C: Collective | 0.11 | .060 | 1.12 | [1.00, 1.25] |
| 5C: Complacency | 0.00 | .970 | 1.00 | [0.89, 1.12] |
| 5C: Constraint | 0.10 | .040 | 1.10 | [1.00, 1.21] |
| 5C: Calculation | 0.01 | .719 | 1.01 | [0.94, 1.10] |
| Vaccine Knowledge | 0.02 | .602 | 1.02 | [0.94, 1.11] |
| Conspiracy Belief | 0.13 | .079 | 1.14 | [0.99, 1.32] |
| Vaccine Danger | -0.08 | .190 | 0.92 | [0.82, 1.04] |
| Science Mistrust | -0.07 | .334 | 0.93 | [0.81, 1.07] |
| Analytic Thinking | 0.04 | .614 | 1.04 | [0.90, 1.20] |
| Disease Vulnerability | 0.06 | .196 | 1.06 | [0.97, 1.15] |
| Self-Other Overlap | 0.03 | .305 | 1.03 | [0.97, 1.09] |
| Conservatism | -0.09 | .003 | 0.92 | [0.87, 0.97] |
| Past Behavior (1=flu shot received) | -0.04 | .686 | 0.96 | [0.78, 1.18] |
| Gender (1=female) | -0.18 | .098 | 0.84 | [0.68, 1.03] |
| Ethnicity (1=non-White) | -0.03 | .826 | 0.97 | [0.75, 1.26] |
| Age | 0.00 | .360 | 1.00 | [0.99, 1.00] |
| Income | 0.20 | <.001 | 1.22 | [1.16, 1.28] |

Note. IRR = Incidence rate ratio. AIC = 6569.25. -2LL = 6491.25. Theta = 0.84.

### COVID-19 Vaccination Intention (Discrete Measure)

As stated in our pre-registration, we estimated an ordinal regression model predicting the discrete COVID-19 vaccination intentions measure including the optimism bias difference scores. Recall that there were three response options for the discrete COVID-19 vaccination intention measure:

A: I would not receive the vaccination even if it’s free ($0).

B: I would receive the vaccination only if it’s free ($0); If I need to pay money, I would not receive the vaccination.

C: I would pay money to receive the vaccination.

We estimated a cumulative logit model with proportional odds predicting the logit of C (willing to pay) vs. A/B (unwilling to pay) and the logit of B/C (willing to vaccinate) vs. A (unwilling to vaccinate). All continuous measures were mean-centered except for the optimism bias difference scores, which have meaningful zero values. Results are shown in Table S11.

Table S11: Cumulative logit model with proportional odds predicting COVID-19 vaccination intention (discrete measure) in Study 2.

|  | **Model 7X** | | | |
| --- | --- | --- | --- | --- |
| **Predictors** | ***b*** | ***p*** | **Odds/**  **Odds Ratio** | **95% CI** |
|  |  |  |  |  |
| Intercept 1 (1=Willing to pay) | -0.28 | .069 | 0.75 | [0.56, 1.02] |
| Intercept 2 (1=Willing to vaccinate) | 1.73 | <.001 | 5.62 | [4.02, 7.86] |
| 5C: Confidence | 0.41 | <.001 | 1.50 | [1.27, 1.76] |
| 5C: Collective | 0.26 | .006 | 1.30 | [1.08, 1.57] |
| 5C: Complacency | 0.01 | .885 | 1.01 | [0.85, 1.21] |
| 5C: Constraint | -0.01 | .869 | 0.99 | [0.85, 1.14] |
| 5C: Calculation | -0.10 | .164 | 0.90 | [0.79, 1.04] |
| Vaccine Knowledge | 0.01 | .847 | 1.01 | [0.88, 1.17] |
| Conspiracy Belief | 0.03 | .779 | 1.03 | [0.83, 1.29] |
| Vaccine Danger | -0.32 | .005 | 0.73 | [0.58, 0.91] |
| Science Mistrust | -0.14 | .268 | 0.87 | [0.69, 1.11] |
| Analytic Thinking | 0.12 | .346 | 1.13 | [0.88, 1.44] |
| Disease Vulnerability | 0.18 | .035 | 1.19 | [1.01, 1.40] |
| Self-Other Overlap | 0.09 | .064 | 1.09 | [0.99, 1.20] |
| Conservatism | -0.12 | .020 | 0.89 | [0.80, 0.98] |
| Past Behavior (1=flu shot received) | 0.74 | <.001 | 2.10 | [1.45, 3.04] |
| Infection Optimism Bias | 0.00 | .888 | 1.00 | [0.99, 1.01] |
| Symptom Optimism Bias | 0.00 | .411 | 1.00 | [0.99, 1.00] |

Note. AIC = 1053.52. -2LL = 1017.52.

### Reasons Influencing COVID-19 Vaccination

Figure S2: Does this reason matter for your decision to vaccinate (Study 2)?


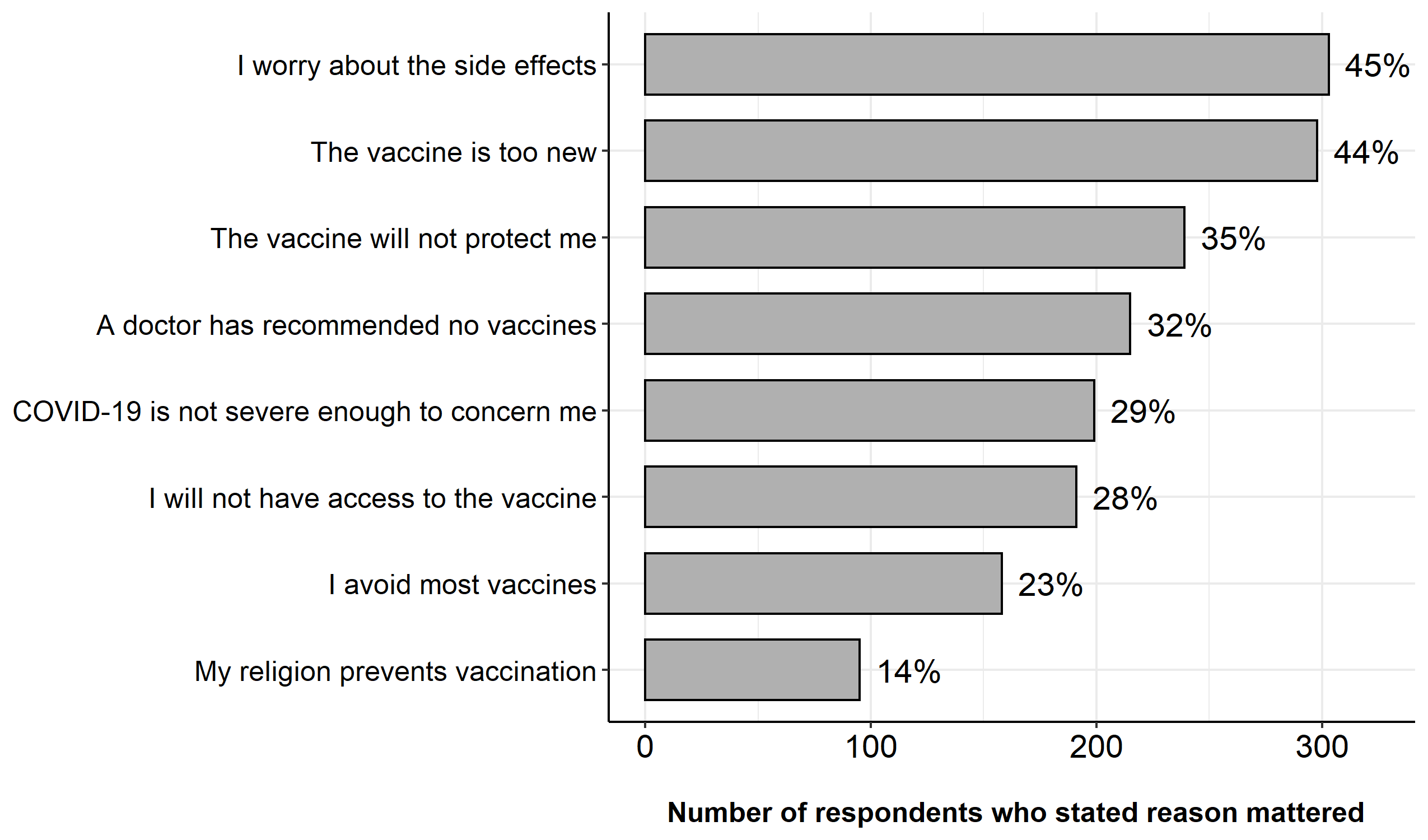


### Social Distancing and Health/Economic Impact of COVID-19

Table S12: Descriptive statistics for supplementary measures and correlations with COVID-19 and dysomeria vaccination intentions in Study 2.

|  |  |  |  | **COVID vaccine** | | **Dysomeria vaccine** | |
| --- | --- | --- | --- | --- | --- | --- | --- |
| **Variable** | ***α*** | **M** | **SD** | **r** | **p** | **r** | **p** |
| Social Distancing | .93 | 5.99 | 1.36 | .34 | <.001 | .40 | <.001 |
| Health Impact | - | 5.73 | 1.44 | .26 | <.001 | .24 | <.001 |
| Economic Impact | - | 6.09 | 1.20 | .06 | .120 | .12 | .001 |
| Heath vs. Economy | - | 3.22 | 1.82 | -.20 | <.001 | -.23 | <.001 |

### COVID-19 Media Consumption

Figure S3: Media consumption and COVID-19 vaccination intention (discrete measure) in Study 2.


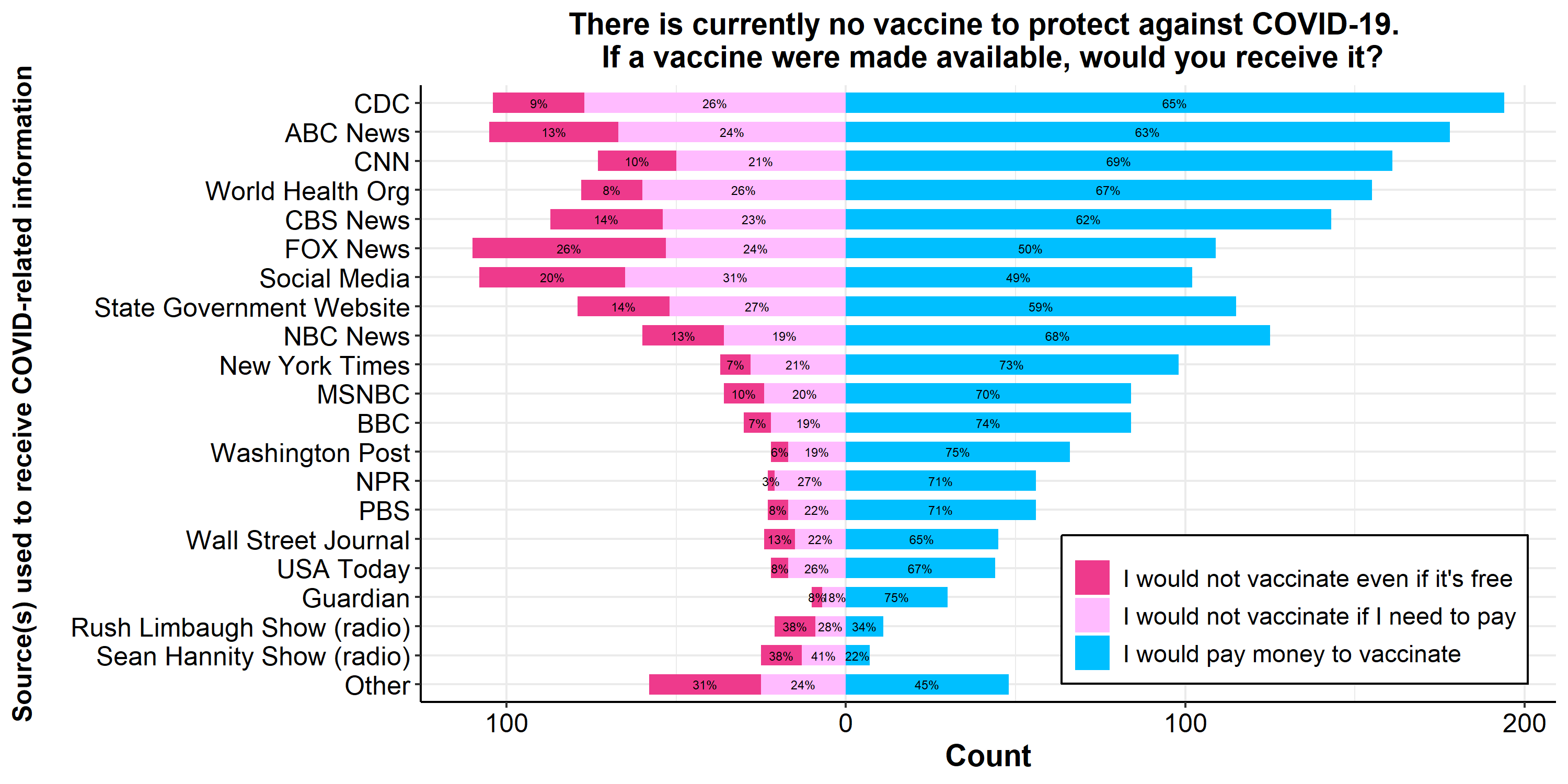


# REFERENCES

Aron, A., Aron, E. N., & Smollan, D. (1992). Inclusion of Other in the Self Scale and the structure of interpersonal closeness. *Journal of Personality and Social Psychology*, *63*(4), 596.

Betsch, C., Schmid, P., Heinemeier, D., Korn, L., Holtmann, C., & Böhm, R. (2018). Beyond confidence: Development of a measure assessing the 5C psychological antecedents of vaccination. *PLoS ONE*, *13*(12). Scopus. https://doi.org/10.1371/journal.pone.0208601

Davis, M. H. (1980). A multidimensional approach to individual differences in empathy. *Catalog of Selected Documents in Psychology*, *10*, 85.

Duncan, L. A., Schaller, M., & Park, J. H. (2009). Perceived vulnerability to disease: Development and validation of a 15-item self-report instrument. *Personality and Individual Differences*, *47*(6), 541–546. https://doi.org/10.1016/j.paid.2009.05.001

Hoerger, M., Quirk, S. W., & Weed, N. C. (2011). Development and validation of the Delaying Gratification Inventory. *Psychological Assessment*, *23*(3), 725–738. https://doi.org/10.1037/a0023286

Jolley, D., & Douglas, K. M. (2014). The effects of anti-vaccine conspiracy theories on vaccination intentions. *PLoS ONE*, *9*(2), e89177. https://doi.org/10.1371/journal.pone.0089177

Nadelson, L., Jorcyk, C., Yang, D., Jarratt Smith, M., Matson, S., Cornell, K., & Husting, V. (2014). I just don’t trust them: The development and validation of an assessment instrument to measure trust in science and scientists. *School Science and Mathematics*, *114*(2), 76–86. https://doi.org/10.1111/ssm.12051

Norris, P., & Epstein, S. (2011). An experiential thinking style: Its facets and relations with objective and subjective criterion measures. *Journal of Personality*, *79*(5), 1043–1080. https://doi.org/10.1111/j.1467-6494.2011.00718.x

Vallacher, R. R., & Wegner, D. M. (1989). Levels of personal agency: Individual variation in action identification. *Journal of Personality and Social Psychology*, *57*(4), 660–671. https://doi.org/10.1037/0022-3514.57.4.660

Weber, E. U., Blais, A.-R., & Betz, N. E. (2002). A domain-specific risk-attitude scale: Measuring risk perceptions and risk behaviors. *Journal of Behavioral Decision Making*, *15*(4), 263–290. https://doi.org/10.1002/bdm.414

1. For Study 1, the reverse-coded item “I am getting vaccinated to protect myself against diseases, not to protect others who are not vaccinated” from Betsch et al.’s (2018) preliminary scale was presented. However, this item had weak correlations with the other two items (*r*s < .09) and therefore was omitted from further analysis. [↑](#footnote-ref-1)
2. This item had weak correlations (*r* < .15) with the other items; it was omitted from further analysis. [↑](#footnote-ref-2)
