# Supplementary material for: Psychological predictors of vaccination intentions among U.S. undergraduates and online panel workers during the 2020 COVID-19 pandemic
Source: PLoS One. 2021 Nov 30;16(11):e0260380. doi: 10.1371/journal.pone.0260380 (PMC8631617; doi:10.1371/journal.pone.0260380)
Supplement: Attachment — Submitted filename: PONE review.docx [file pone.0260380.s002.docx]

Thank you for the opportunity to review this manuscript. I appreciate the amount of work that has been put into this study; the authors should be commended for their efforts to study the psychological roots of vaccine hesitancy during the current pandemic. Below are my concerns

- I have major problems with the way the data are presented, and the manuscript is written and I recommend major revision to have a more concise manuscript with a clear flow and more clear conclusion. I include a couple of examples on issues that need to be further clarified below.
- I do not see any benefit of splitting the study samples into 1& 2, and if there was, it was not made clear in the presentation of the study. I understand that sample 2 was needed to have a more age-diverse sample, but why not combine them? If it was because you expected differences between the two samples, then explain why you would put this assumption? On what basis? This needs to be explained or changed. There are also differences in the questions asked to each sample which were not explained; on what basis did the author choose to do so? Any benefits? Wouldn't that increase variability of the results among the samples?
- The manuscript reads more like a thesis than a concise manuscript it lacks “straight to the point” presentation of data and clear conclusions. I recommend rewriting the manuscript, especially the introduction, which should be one coherent piece of work, also the methods and the results. The conclusion in the abstract will need reconsideration as it starts with a vague sentence and does not give any concise conclusion
- The questionnaires used are very lengthy, and despite the attention checks the authors have described, no information has been provided regarding how many participants were excluded due to failing the attention check; they only report a general total number of 38 excluded for different reasons for sample one and same regarding sample 2 were 172 were excluded for several reasons.
- Line 85: "Whereas previous research has examined attitudes about vaccination 86 for diseases that are hypothetical (e.g., Haase et al., 2015; Jolley & Douglas, 2017), relatively 87 low-threat (e.g., seasonal flu; Zhang et al., 2010) or mostly eradicated in the U.S. (e.g., Dubé et 88 al., 2015), the present research investigates vaccination intentions during a unique period of uncertainty involving a highly contagious disease when effective treatments or vaccines had not yet been developed." The authors should report the published evidence on vaccine intentions in the COVID-19 era
- The authors mention that” Sample 1 included 346 undergraduate students from the University of Illinois at Urbana183 Champaign recruited in exchange for course credit” Can you elaborate more on what do you mean here? Were the participants given a choice to join? How did you guarantee that participants were aware that their participation is optional?
- The discussion section can be improved by highlighting the take-home message from the study and take the reader through what has been done and what does that mean rather than displaying the results. As an example in line 418: "knowledge, no prior study has simultaneously measured vaccine attitudes for both a hypothetical disease and a real disease during a pandemic. Measuring both together and determining which variables predict well across measures is important, as it provides context for past work and can bolster confidence in how well past research applies to actual intentions during a crisis period…… The current work shows that factors associated with immunization decisions for a hypothetical disease are also mostly predictive of real vaccination intentions." I do not see the point here? Can you elaborate on the reasons behind studying vaccine attitudes for both hypothetical disease and a real disease during a pandemic? Not at any point of the manuscript, this was discussed clearly
- The tables are confusing with too many details; I suggest editing them to highlight the most important message from each table and move any unnecessary information to the supplementary file.
- In the supplementary file, Why was the attention check different between samples 1 &2? What did you base this on?

For Sample 1, the following item was asked prior to the demographics questionnaire:

- Thank you for your responses. You are almost finished with this study. Before answering a few questions about yourself, we have one last question. Do you think we should include your responses in our study? That is, did you take the study seriously and respond thoughtfully? Your credit assignment for this study does not depend on your response to this question. [Yes, I responded thoughtfully / No, I did not respond thoughtfully.]

Sample 2 Only

For Sample 2, the following item was embedded in the optimism bias measure:

| If you are reading this, please select 67 on  this sliding scale. |  |
| --- | --- |
